# Bibliography

**Published:** 1898-06

**Authors:** 


### Bibliography.

Annual and Analytical Cyclopædia of Practical Medicine. By Charles E. de M. Sajous, M.D., and one hundred Associate Editors, assisted by Corresponding Editors, Collaborators, and Correspondents. Illustrated with Chromo-Lithographs, Engravings, and Maps. Volume I. Philadelphia, New York, and Chicago: The F. A. Davis Company, Publishers, 1898.

Dr. Sajous and his collaborators, together with the essential aid of the publisher, have produced a work of inestimable value to medical practitioners, and in this class must be included all specialists of the healing art. The debt which the profession owes Dr. Sajous for his untiring labors in bringing this work, in its condensed form, to their attention, and thus relieving the busy and overworked practitioner, may never be paid, but it certainly must be appreciated, and should be fully recognized.

The editor says in his preface, "The work, when completed, will present all the general diseases described in text-books on practical subjects,—medicine, surgery, therapeutics, obstetrics, etc.,—and inserted in their logical order in the text all the progressive features of value presented during the last decade. This will remove the cause of dissatisfaction caused by the absence of general subjects in the older work. If the year brings forth nothing new upon any particular disease, the latter will, at least, appear as it was when last studied, whether this be one, two, five, or twenty years before. The general arrangement adopted will make it possible to cover the entire field in six volumes."

The subjects are arranged in alphabetical order, and are treated in an exhaustive manner. The article on "Abdominal Injuries" contains one hundred article excerpts, besides the general text. This article opens the book under A. This letter covers the larger portion of the volume, or five hundred and thirty pages. The balance is made up under the letter B. These pages are arranged in double columns, the text in clear type, cases and literature in smaller type. The arrangement seems to the writer particularly effective for study. In illustration, the portion devoted to *abscess.* The derivation is given followed by a full definition. This is succeeded by "varieties, etiology, pathology, location (organ or tissue involved), symptoms, differential diagnosis, prognosis, treatment," followed by external remedies, surgical measures, and full excerpts from the literature of the subject.

The therapeutics of this volume forms an especially valuable part of it, including many recently introduced remedies, at the same time discarding many obsolete agents.

While the scope of the work covers all that has been found valuable in medicine for a series of years, and does not, perhaps, enter directly into the practical work of dentistry, it is nevertheless very important for the members of this specialty; in fact, when complete, it will be an entire library to which they can refer upon all occasions, and will lead to the avoidance of error in quoting, an unfortunate tendency with many young writers. The work can, therefore, be recommended without reserve as altogether the most complete and satisfactory cyclopædia within the knowledge of the writer.

Descriptive Anatomy of the Human Teeth. Fourth Edition. By G. V. Black, M.D., D.D.S., Sc.D. The S. S. White Dental Manufacturing Co., 1897.

There is no better evidence of the value of a book than the successive editions called for, and this, the fourth edition of this valuable work, is satisfactory proof, if any were needed, that it has filled a prominent place in dental teaching, and continues to hold the position occupied by the first edition.

The subject-matter of the volume was so thoroughly gone over in preceding editions that there has been but slight changes demanded for this, the fourth, and these "consist in the introduction of tables of the angles of teeth, intended to aid the student in fixing these in his mind and in the introduction of the word *embrasure* as an additional technical term." This addition, though covering but limited space, is especially valuable, as it will clear up in the minds of readers a somewhat obscure matter and lead, it is hoped, to more definite descriptions by writers. It is questionable, however, whether the introduction of the word *embrasure*, while correctly applied, will tend to give any clearer idea of the space between the proximal surfaces widening towards the buccal and lingual surfaces. The tendency to increase the terms used in dentistry is not to be commended, and while the motive is good, the practice is leading to great confusion in descriptive work.

The book has been so carefully reviewed in previous editions and has been so fully appreciated that it seems unnecessary to say more than that it will, probably, remain for the future, as it has in the past, the most satisfactory work upon the anatomy of the human teeth, and there does not seem any reason to expect that it can ever be superseded as a text-book.

A Text-Book of Dental Pathology and Therapeutics, including Pharmacology. By Henry H. Burchard, M.D., D.D.S. Philadelphia and New York: Lea Brothers & Co., 1898.

This work, by Dr. Burchard, was placed in the hands of the editor too late for review in the present number. A cursory examination of its contents leads to the opinion that it will fill a place not heretofore occupied, but whether it will meet the expectations of workers upon similar lines of practice must be left for decision through more careful reading.

A full review will be given in the July number of this journal.

